# Pulsed operation of perovskite LEDs: a study on the role of mobile ions

**DOI:** 10.1093/nsr/nwae128

**Published:** 2024-03-29

**Authors:** Miguel A Torre Cachafeiro, Naresh Kumar Kumawat, Feng Gao, Wolfgang Tress

**Affiliations:** Institute of Computational Physics, School of Engineering, Zurich University of Applied Sciences (ZHAW), Winterthur 8400, Switzerland; Institut des Matériaux, École Polytechnique Fédérale de Lausanne (EPFL), Lausanne 1015, Switzerland; Department of Physics, Indian Institute of Technology Indore, Indore 452 020, India; Department of Physics, Chemistry and Biology (IFM), Linköping University, Linköping 583 30, Sweden; Department of Physics, Chemistry and Biology (IFM), Linköping University, Linköping 583 30, Sweden; Institute of Computational Physics, School of Engineering, Zurich University of Applied Sciences (ZHAW), Winterthur 8400, Switzerland

**Keywords:** perovskite, light-emitting diode, pulsed operation, simulation, transient electroluminescence

## Abstract

Metal halide perovskite light-emitting diodes (PeLEDs) are a promising technology for energy-efficient and cost-effective lighting and displays, thanks to their tunable color emission, high brightness, color purity and low-temperature fabrication. However, the mixed ionic-electronic conductivity of perovskite materials presents unique challenges, as ionic defects can redistribute under operation, affecting the energy landscape and charge recombination mechanisms. Our drift-diffusion simulations establish a connection between the transient electroluminescence (TrEL) signals of PeLEDs under pulsed operation and the influence of mobile ions. We find that the TrEL plateau value’s dependence on the duty cycle and end-of-pulse overshoot can be explained by the time-varying distribution of ionic defects. The inclusion of mobile ions is crucial to understand the TrEL response. Moreover, the simulations highlight injection barriers at the perovskite/charge-transport layer interfaces, such as is the case for the hole transport layer in our example, as a significant source of non-radiative charge recombination. These findings contribute to the understanding of transient ionic processes in perovskite-based devices.

## Teaser

This study explores ionic redistribution in perovskite LEDs during pulsed operation, using drift-diffusion simulations to analyze the impact of mobile ionic defects on light emission and providing insights into the functionality of these promising devices.

## INTRODUCTION

Lighting represents a significant portion of global electricity consumption; hence, there is a continuous need to explore new technologies that are both energy-efficient and cost-effective. In this context, perovskite light-emitting diodes (PeLEDs) have emerged as a promising technology, offering high color purity, high brightness, tunable color emission and low-cost fabrication processes [1,2]. Halide perovskite materials have also attracted significant attention as promising candidates for next-generation perovskite solar cells (PSCs) and photodetectors [3,4]. One important property of perovskite semiconductors is their mixed ionic-electronic conductivity, which refers to their ability to conduct both ionic matter and electronic charges simultaneously [5]. This property has significant implications for the performance and stability of optoelectronic devices based on halide perovskites, and, therefore, extensive research efforts have been focused on understanding and controlling the effects of the mixed ionic-electronic conductivity in these materials [6,7].

For practical applications, the transient response of mobile ions can become a critical factor, particularly under pulsed operation with pulse width modulation (PWM). Ionic defects in perovskite-based devices can slowly redistribute under bias or illumination, in timescales ranging from milliseconds to minutes or even hours [8]. If the mobile ion density is high enough, redistribution can significantly change the energy landscape in the device and shift the balance between different charge recombination mechanisms [9], affecting device performance and stability. In a recent study by Kumawat *et al.* [10], the transient electroluminescence (TrEL) signal of formamidinium lead triiodide (FAPbI_3_ or FAPI) near infrared (NIR) PeLEDs was measured under different pulsed frequency ranges and varying pulse widths. The measured TrEL signal exhibited duty-cycle-dependent features that were attributed to the characteristic response times of mobile ions. A rather constant plateau value of the TrEL was found after turning on, which unexpectedly showed higher intensity for higher duty cycles. Furthermore, a large overshoot was observed at the end of the voltage pulse, which decreased and eventually disappeared with increasing duty cycle. Such large overshoots in the TrEL have also been previously reported for PeLEDs and PSCs and linked to the distribution of mobile ions [11,12].

The study conducted by Kumawat *et al.* [10] established a connection between the distinct TrEL behavior of PeLEDs and the influence of mobile ions. While a mathematical model and schematic were presented, a comprehensive understanding requires physical simulations. We here present drift-diffusion simulations using a full device model. The obtained results validate the significance of ionic redistribution in determining the TrEL characteristics of PeLEDs, offering further insights into the underlying mechanisms.

## MATERIALS AND METHODS

**Figure 1.**
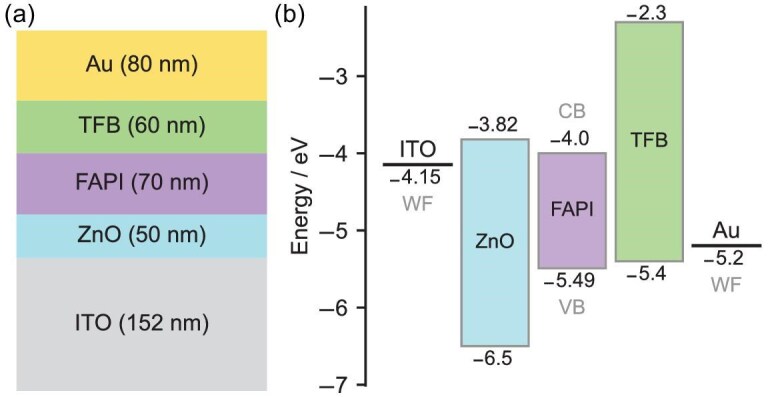
PeLED model device for drift-diffusion simulations. (a) Material stack and layer thicknesses. (b) Energy-level diagram. WF indicates the work function of the electrodes; CB and VB indicate the energy of the band edge for the valence band and conduction band, respectively.

The drift-diffusion model used in this study is implemented in the Setfos 5.4 software package. The model takes into account the transport of electrons and holes, as well as mobile ions [23]. The device structure considered in the simulation is a PeLED consisting of a perovskite emissive layer sandwiched between charge selective contacts; an electron transport layer (ETL) and a hole transport layer (HTL). The model device is designed to closely mimic the real device, using optical and electrical parameters of the actual materials employed, wherever possible. As depicted in Fig. 1, the model device is composed of indium tin oxide (ITO)/zinc oxide (ZnO)/FAPI perovskite/poly(9,9-dioctyl-fluorene-co-N-(4-butylphenyl)diphenylamine) (TFB)/Au, which is consistent with the experimental device [10]. In practice, a thin MoO_3_ layer is added between the Au and TFB HTL to facilitate hole injection [24] and a thin polyethylenimine layer is deposited on top of ZnO to passivate its surface and reduce the effective work function of the ZnO [25]. However, it is important to note that the model is limited to general and planar layers, which restricts its ability to fully represent the effects of treatments, additives or variations in crystal sizes (e.g. ZnO nanocrystals, ODEA (2,2-(oxybis(ethylenoxy)) diethylamine) additive in perovskite, etc.). The electrical properties of the materials used in the simulation are shown in Table 1. Charge mobilities have been selected to remain within the range of reported values for ZnO [26,27], FAPI [15] and TFB [16]. The energy levels chosen are based on values reported for this device architecture [10,28]. The work function of ITO has been lowered to provide a contact with sufficient charge injection in the simulations. A 50-Ω series resistance is added to the model to account for the measuring resistor used in the experimental setup [10].

**Table 1.** Material parameters used for simulation.

Parameter	TFB	FAPI	ZnO
Thickness	60 nm	70 nm	50 nm
VB energy	5.4 eV [10]	5.49 eV [13]	6.5 eV
CB energy	2.3 eV [10]	4.0 eV [13]	3.82 eV [10]
Density of states VB	1 × 10^20^ cm^−3^	1 × 10^19^ cm^−3^ [14]	1 × 10^20^ cm^−3^
Density of states CB	1 × 10^20^ cm^−3^	1 × 10^19^ cm^−3^ [14]	1 × 10^20^ cm^−3^
Electron mobility	1 × 10^−7^ cm^2^ (V s)^−1^	0.5 cm^2^ (V s)^−1^ [15]	0.0125 cm^2^ (V s)^−1^
Hole mobility	2 × 10^−4^ cm^2^ (V s)^−1^ [16]	0.5 cm^2^ (V s)^−1^	1 × 10^−6^ cm^2^ (V s)^−1^
Bimolecular recombination prefactor	–	1 × 10^−10^ cm^3^ s^−1^ [17]	–
Dielectric constant	3.5 [18]	20 [19]	12 [20]
Cation density	–	5 × 10^17^ cm^−3^ [21]	–
Anion density	–	5 × 10^17^ cm^−3^ [21]	–
Cation mobility	–	1 × 10^−9^ cm^2^ (V s)^−1^	–
Anion mobility	–	Static	–
Bulk trap densities	–	1 × 10^17^ cm^−3^ [22]	–
Bulk trap depth	–	0.2 eV	–
Capture rates	–	2 × 10^−11^ cm^−3^s^−1^	–
Interface recombination velocity	0.75 cm s^−1^	–	0.75 cm s^−1^
Device area		7.25 mm^2^	
Series resistance in circuit		50 Ω	

At both interfaces of the perovskite layer, non-radiative Shockley–Read–Hall (SRH) recombination can take place. The model used for SRH recombination at the interfaces is explained in the online supplementary material. In the perovskite bulk, a uniform distribution of shallow trap states is included [29]. Bulk traps do not participate in recombination, since most deep ones have been found to be located close to the interfaces [30]. The emission calculation is directly coupled to the generated photons from band-to-band recombination in the bulk perovskite, where only this type of charge recombination is allowed.

Halide perovskite materials (ABX_3_) can contain various types of mobile ions, and there is no clear consensus on the dominant species. Previous studies have suggested the presence of halide vacancies (V$_{\rm X}^{+}$ cations), potentially compensated by organic or metallic cation vacancies (V$_{\rm A}^{-}$ or V$_{\rm B}^{-}$ anions), and interstitial halides (X$_{\rm i}^{-}$ anions) [31]. Recent analytical models propose a scenario where only cations can redistribute, while compensating anions remain immobile [21]. Therefore, for our simulations, we consider two mobile ions with opposite charges, both exhibiting identical concentrations, but where only cations can move to screen the electric field and anions are kept fixed in a uniform distribution throughout the perovskite layer. However, since anions (e.g. I$_{\rm i}^{-}$) may actually migrate as well [28,32,33], it was crucial to investigate whether the observed trends persist when considering both types of ionic charges as mobile. For the base case, only the cation species are mobile, and the anions act as a stationary counter charge. This is then later compared to the case where both types of ionic charges are mobile. Mobile ions in the simulation cannot act as traps or recombination centers themselves; they do not interact with electrons and holes, but only change the energy landscape by influencing the electric field. Ionic mobility is restricted to the perovskite bulk. For the same PeLED device architecture studied here, previous reports have shown migration of I^−^ anions into and through the TFB HTL during device operation, using time-of-flight secondary ion mass spectrometry measurements [33]. Anion penetration into the HTL has been identified as a main source for performance decrease and device degradation, where some of the losses can be reversed upon rest as anions back diffuse into the bulk perovskite. For our drift-diffusion simulations, we assume that the PeLED is being operated prior to any degradation caused by anion diffusion into the HTL. Ion densities are set to 5 × 10^17^ cm^−3^, based on values reported for halide perovskite thin films [21,34]. For mobile cations, a mobility of 1 × 10^−9^ cm^2^ V^−1^s^−1^ is set, which remains within the wide range of reported values [35,36]. As a basic validation of the model and parameters used, a current density–voltage (J-V) curve is shown in Fig. S1 within the online supplementary material, comparing the measured and simulated performance.

## RESULTS AND DISCUSSION

**Figure 2.**
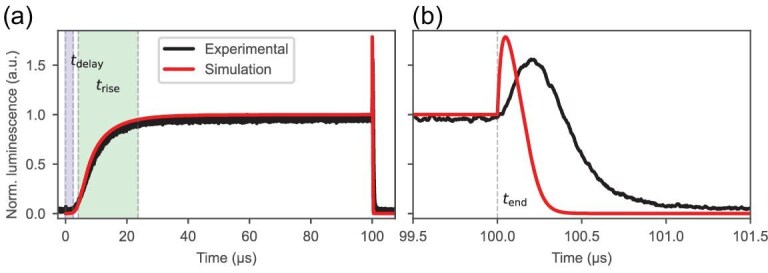
Comparison between simulated and experimental TrEL signals for a single 100-*μ*s pulse at 1.7 V, normalized at the plateau value. (a) Full TrEL signals. (b) Overshoot at the end of the pulse. For the purpose of comparison, the experimental 100-*μ*s pulse recorded at a 10% duty cycle (1 kHz) is used, where the measured pulse shape in its converged state is expected to closely resemble that of the first pulse.

The TrEL behavior for microsecond-long pulses for both PSCs and PeLEDs has been previously studied, revealing shared characteristics and a noteworthy phenomenon upon bias removal: a high and fast overshoot [10–12]. Furthermore, the measured TrEL signals exhibit a delay time before response, followed by a rise time to a plateau value, and ultimately the mentioned overshoot at the end of the voltage pulse, as depicted in Fig. 2, which presents a comparison between experimental and simulated TrEL signals for a single 100-*μ*s pulse at 1.7 V. In our model, the delay time has been adjusted by tuning the hole mobility in the HTL, whereas the rise time is ruled by the filling of shallow trap states in the perovskite bulk. As can be seen in Fig. 2(b), the overshoot characteristics are significantly different in terms of timescales, which may be due to differences in the choice of charge mobility parameters. However, the focus of the study is not to fit the shape of the overshoot, but rather to understand how and why it scales with varying duty cycles.

**Figure 3.**
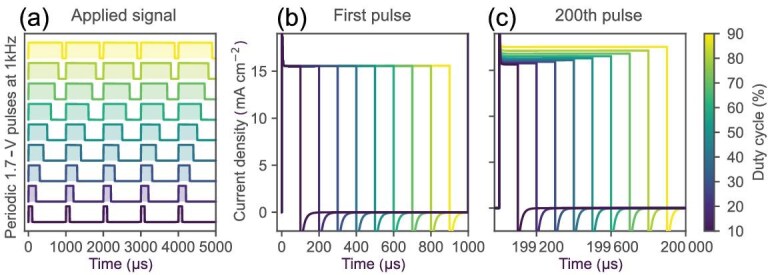
(a) Applied voltage signal in the simulations, consisting of periodic 1.7-V pulses at 1 kHz with varying duty cycles. (b) Simulated current density response of the device for the first pulse. (c) Simulated current density after 200 consecutive pulses.

To investigate the impact of the duty cycle on the pulsed operation of the PeLED device, it is crucial to look at the cumulative effect of ionic redistribution. Figure 3(a) depicts the continuous train of voltage pulses at 1 kHz and different duty cycles used for the simulations, which were carried out up to 200 pulses. Panels (a) and (b) of Fig. 3 show the device current response for the first and the 200th pulses, respectively. The simulated transient current (TrJ) response shows the same trend as the experimental one [10], with higher duty cycles resulting in slightly higher current densities.

**Figure 4.**
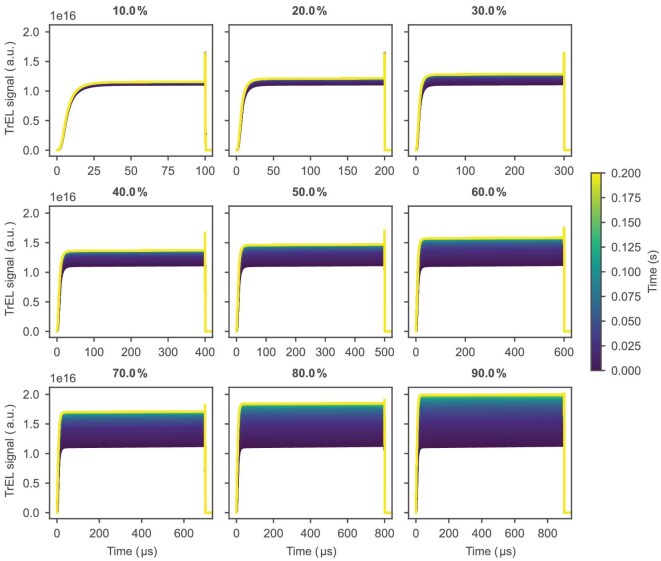
TrEL evolution during 200 1.7-V pulses at 1 kHz (0.2 s total) for different duty cycles (%). The TrEL signal plotted corresponds to the generated photons from bimolecular recombination, and hence the order of magnitude.

**Figure 5.**
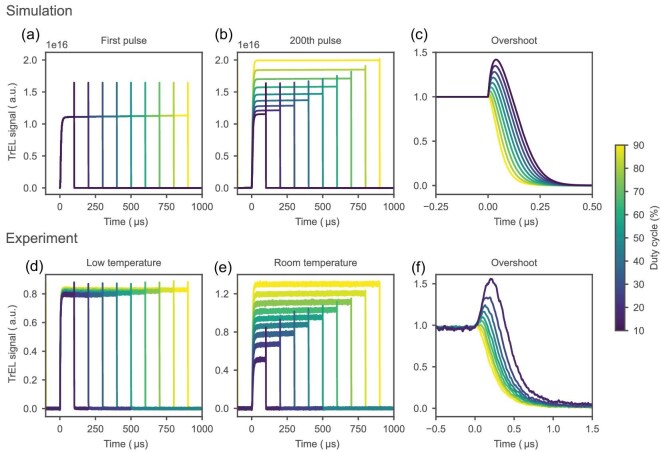
Simulated (a–c) and experimental (d–f) TrEL results for a 1-kHz signal with varying duty cycles. (a) First simulated pulse, starting from rest. (b) Simulated TrEL signals for the converged-state pulse after 0.2 s (200th pulse). (c) Simulated overshoot with normalized plateau value for the 200th pulse. (d) Experimental low-temperature TrEL signal measured at 175 K and 3.5 V. (e) Experimental TrEL signal at 1.7 V measured at room temperature. (f) Experimental overshoot with normalized plateau value.

Figure 4 and Fig. S2 illustrate the simulated temporal evolution of the TrEL signal shape for 200 consecutive voltage pulses at 1 kHz and 1.7 V, with varying duty cycles from 10% to 90%. The time taken to reach a converged signal (Fig. S3) is determined by the ion mobility. At 1 kHz, the increase in TrEL during a single voltage pulse is not observable; the TrEL plateau looks relatively flat for all pulses. However, the cumulative effect due to ionic response at longer timescales becomes visible in the simulation of the pulse train. In Fig. 5, a comparison is presented between the first (Fig. 5(a)) and last (Fig. 5(b)) simulated pulses for different duty cycles. Initially, all signals exhibit a similar shape, but diverge and converge differently depending on the duty cycle. To look at the first pulse in the experiment, where the plateau has not yet scaled due to ionic redistribution, the TrEL signal was measured at a lower temperature of 175 K (Fig. 5(d)), which allows us to look at the state where mobile ions are not able to redistribute since they remain mostly ‘frozen’ in their initial position. The overshoot at low temperature appears smaller and mostly constant, due to ionic redistribution from earlier measurements taken at higher temperatures. At room temperature after ionic redistribution, higher duty cycles lead to a higher plateau value, accompanied by a reduction of the overshoot.

In agreement with the experimental results (Fig. 5(e)), the simulated profiles show a direct scaling of the plateau value and overshoot height with increasing duty cycle. The TrJ and TrEL scale in the same direction but with different magnitudes, both in simulations and in experiments. The larger increase of the EL compared to the current indicates that the ionic response not only results in a difference in injected charge density, but also in a shift between different charge recombination mechanisms, resulting in an enhanced radiative yield with the duty cycle, as demonstrated in Fig. S9. The slight increase of the TrJ can be explained by a higher electric field in the charge transport layer (CTLs) upon ionic screening of the electric field in the perovskite layer, as shown in Fig. S4. The higher field in the CTLs facilitates charge carrier injection, since energy barriers and mobilities of the CTLs are the limiting factors for the injection current in our device.

**Figure 6.**
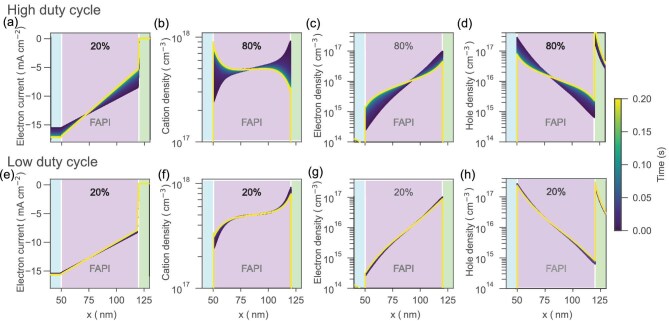
Temporal evolution of charge density profiles for a simulated 1-kHz signal at 1.7 V for 0.2 s (200 pulses). Dark blue color indicates the initial state and yellow indicates the final state. Electron current profiles (a and e) and charge density profiles of cations (b and f), electrons (c and g) and holes (d and h) across the perovskite layer width (x). (a–h) The temporal evolution for an 80% duty cycle (a–d) and a 20% duty cycle (e–h). ETL (blue) and HTL (green) are left and right of the FAPI layer, respectively.

For the temporal evolution to occur, the inclusion of mobile ions in the simulation is strictly necessary. Without mobile ions, the simulated TrEL signal remains periodic from the start. In contrast, when mobile ions are included, their distribution varies based on the duty cycle, which determines the ratio between on and off times of the applied voltage. Figure 6 shows the evolution with time of the charge density profiles across the perovskite layer during the voltage-on time for the entire transient simulation of 200 pulses at 1 kHz (0.2 s) depicted in Fig. 4. Two cases are shown for comparison, a high duty cycle (80%; top row) and low duty cycle (20%; bottom row). Figure 6(a) shows the electron current profiles across the perovskite layer and interfaces with the ETL and HTL. As shown in Fig. 6(b), initially, cations accumulate close to the HTL, due to the built-in field of the device. Under the applied bias, the accumulated mobile ions shift from one interface to the other, when given enough time. Such redistribution leads to a screening of the electric field across the perovskite, which in turn changes the energy landscape in a way that favors higher radiative recombination in the bulk. As the field is screened, electron and hole accumulation close to the interfaces is reduced, as illustrated in Fig. 6(c)–(d). The increased overlap of electron and hole densities in the bulk results in higher band-to-band recombination during the voltage pulse, with lower SRH recombination at the interfaces. In particular, as seen in Fig. 6(a), non-radiative recombination at the interface with the HTL dominates—almost no current drops at the interface with the ETL, yet a significant amount does at the HTL interface. This is due to the injection barrier at the HTL, which results in a higher hole concentration on the HTL compared to the perovskite. This situation provides a pathway for the electrons accumulated in the perovskite to efficiently recombine at the perovskite/HTL interface. In Fig. 6(a), the slope in the electron current along the width of the FAPI layer indicates the amount of injected charges that recombine radiatively. For the high duty cycles, the slope becomes steeper along the perovskite layer and less current is lost non-radiatively at the HTL interface. The finding of non-radiative recombination dominating at the HTL interface is in agreement with a previous study for the same type of NIR PeLED [28].

**Figure 7.**
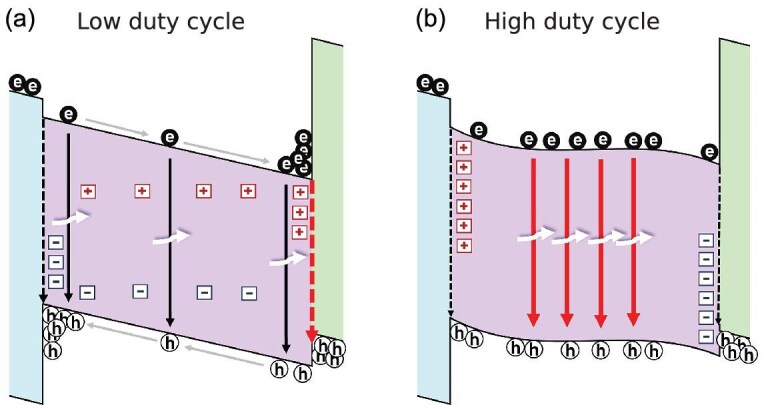
Band diagrams showing a low duty cycle scenario (a) where mobile ions ([+] and [-]) are still close to their initial distributions and a high duty cycle scenario (b) where ions have redistributed. The simulated band-edge profiles are shown in Fig. S4. ETL and HTL are left and right of the FAPI layer, respectively.

The TrEL signal exhibits an overshoot when, during the voltage pulse, there is a high accumulation of electrons near the HTL and holes near the ETL. Before ions redistribute, injected electronic charges quickly drift through the perovskite and accumulate close to the interfaces, limited by the amount of surface recombination. Figure 7(a) illustrates this scenario, corresponding to a low duty cycle where non-radiative recombination at the HTL interface is significantly high. When the voltage is turned off, the accumulated charges move rapidly into the bulk and recombine radiatively, resulting in a rapid and high overshoot in the TrEL. Conversely, in a high duty cycle scenario, as depicted in Fig. 7(b), where mobile ions have redistributed and the HTL side becomes depleted of cations, the potential becomes flatter across the perovskite. This leads to less accumulation of electrons and holes near the interfaces during the voltage pulse (Fig. 6(c)–(d)) and, consequently, no overshoot in the TrEL when the voltage is turned off. This is accompanied by a scaling of the TrEL plateau with the duty cycle, which is dominated by the shift in the balance between non-radiative recombination at the HTL interface and radiative recombination in the perovskite bulk. In contrast to schematic explanations provided elsewhere where a symmetric scenario is depicted [1,10], the drift-diffusion results presented here show that the interface with an injection barrier (here the perovskite/HTL interface) dominates the shift between different types of recombination mechanisms as mobile ions respond.

The obtained results from the transient drift-diffusion simulations for hundreds of voltage pulses can be approximated by a simple shortcut. Since mobile ions do not have enough time to respond within the time frame of a single microsecond-long pulse, an initial distribution can be calculated and then kept fixed for the transient simulation (Fig. S5). By calculating the ionic distribution using different precondition voltage levels, the effect of the duty cycle can be closely approximated, as shown in Figs S6 and S7, where the scaling with varying ionic precondition voltages is compared for different interface SRH recombination velocities; a sufficiently high interface recombination velocity at the HTL interface is needed to achieve the larger scaling of the plateau value. This may prove useful to further analyze the effect of varying parameters in a PeLED in order to limit or exploit duty cycle dependencies.

Similar to what has been found before for PSCs [37,38], the interfacial recombination current increases with increasing injection barrier height, as shown in Fig. S10. A high-enough level of non-radiative interface recombination with a strong dependence on the ionic distribution is key to observe the trends seen in experiments, since otherwise the TrEL simply scales due to the differences in injected current density, resulting in an unaltered radiative yield. This effect is qualitatively independent of a change in the built-in potential as probed by variations of the metal work function in Fig. S10.

Furthermore, as can be seen in Fig. S8 for the case where both cations and anions are mobile, the results exhibited a slightly increased scaling of the TrEL plateau signal with the duty cycle. However, the observed trends remained consistent with the use of one mobile ion (only mobile cations). Regardless of the employed model (one or two mobile ions), the electric field is effectively screened by the ionic redistribution, and the TrEL properties exhibit the same dependencies.

Varying the perovskite layer thickness does not qualitatively change the results (Fig. S11). The share of interfacial recombination decreases for thicker layers independent of the ion distribution. The reason is an increased probability for band-to-band recombination due to the increased travel distance and reduced drift velocity of electronic charges caused by a reduced electric field for the same potential (Fig. S12).

Reducing non-radiative losses through defect states at the interfaces continues to be important for PeLED device engineering; interface recombination can be more severe when an energy-level mismatch resulting in an injection barrier is present. The degree to which ionic redistribution affects recombination processes in PeLEDs can be understood from PWM operation; our simulations point out that reducing non-radiative recombination at interfaces, either by defect passivation or improved energy-level matching, should result in a decreased TrEL scaling with the duty cycle.

## CONCLUSION

This study has presented an explanation for the TrEL characteristics of PeLEDs during pulsed operation, utilizing transient drift-diffusion simulations. The scaling with the duty cycle of the TrEL plateau value during the voltage pulse and the overshoot at the end of the pulse can be explained by the distribution of ionic defects dependent on the duty cycle. Considering the properties of these defects is essential for the design and advancement of PeLED-based technologies, as they significantly impact their luminescent properties. Notably, the inclusion of mobile ions into the simulation is crucial to accurately explain the experimental results, as without them, the TrEL signal remains periodic. For isolated voltage pulses, ionic transients are not observable in the kilohertz frequency range, but the pulse train’s cumulative effect leads to varying but approximately static ionic distributions after a sufficient number of pulses. Moreover, interfacial recombination plays a vital role as it affects the scaling with the duty cycle of the TrEL plateau value. The simulations conducted here validate the theoretical models proposed in previous studies. Additionally, they identify the importance of injection barriers to the CTLs. In the simulated stack based on an experimental realization with FAPI and TFB, they point towards the perovskite/HTL interface as the main competing source of non-radiative interfacial SRH recombination. Drift-diffusion simulations including mobile ionic charges continue to be a useful tool for better understanding complex transient processes in perovskite-based devices.

## Supplementary Material

nwae128_Supplemental_File
