# Supplementary material for: Pulsed operation of perovskite LEDs: a study on the role of mobile ions
Source: Natl Sci Rev. 2024 Mar 29;12(5):nwae128. doi: 10.1093/nsr/nwae128 (PMC11970239; doi:10.1093/nsr/nwae128)
Supplement: nwae128_Supplemental_File [file nwae128_supplemental_file.pdf]

Supplementary Information for **Pulsed Operation of Perovskite LEDs: A Study on the Role of Mobile Ions**

Miguel A. Torre Cachafeiro[1,2,*], Naresh Kumar Kumawat[3,4], Feng Gao[4], Wolfgang Tress[1,*]

[1]Institute of Computational Physics, School of Engineering, Zurich University of Applied Sciences (ZHAW), Winterthur, Switzerland
[2]Institut des Matériaux, École Polytechnique Fédérale de Lausanne (EPFL), Lausanne, Switzerland
[3]Department of Physics, Indian Institute of Technology Indore, Indore, India
[4]Department of Physics, Chemistry and Biology (IFM), Linköping University, Sweden

***Corresponding authors.** Email: miguel.torre@zhaw.ch, wolfgang.tress@zhaw.ch

June 11, 2024

Recombination models

The transient electroluminescence (TrEL) signal originates from light emission of the radiative recombination of injected electrons and holes. Bimolecular recombination, i.e. direct band-to-band recombination, is used to directly compute the rate of generated photons. This quantity is directly used to analyse the simulated TrEL signals - plotted in the main report in arbitrary units. The bimolecular recombination rate is given by:

$$R_{bimolecular} = \beta(np - n_i^2) \tag{1}$$

where β is the radiative recombination coefficient (cm^{-3}s^{-1}), n is the density of electrons and p the density of holes. The rate of non-radiative (SRH) recombination through energetic defect states at the interfaces is modelled by:

$$R_{SRH_{int}} = q\frac{np - n_{i,int}^2}{\frac{1}{v_p}(n + \hat{n}) + \frac{1}{v_n}(p + \hat{p})} \tag{2}$$

where v_p and v_n are hole and electron recombination velocities (cm s^{-1}), respectively. The parameters $\hat{n}$ and $\hat{p}$ define the dependency of the recombination rate on the energy level of the interface defect $E_{t,int}$, which is set to the mid-energy between the minimum of CB levels and the maximum of VB levels left and right of the interface:

$$\hat{n} = N_{0,n} \exp \frac{E_{t,int}}{kT} \tag{3}$$

$$\hat{p} = N_{0,p} \exp \frac{E_{t,int}}{kT} \tag{4}$$

with $E_{t,int}$ being -0.745 eV for the perovskite/ETL interface and -0.7 eV for the perovskite/HTL interface. $N_{0,n}$ and $N_{0,p}$ are the densities of states in the CB and VB, respectively.

Supplementary Figures

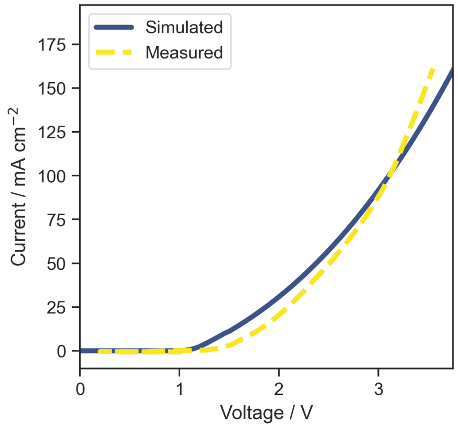

Figure SI 1: J-V curves for simulated and measured PeLED device.

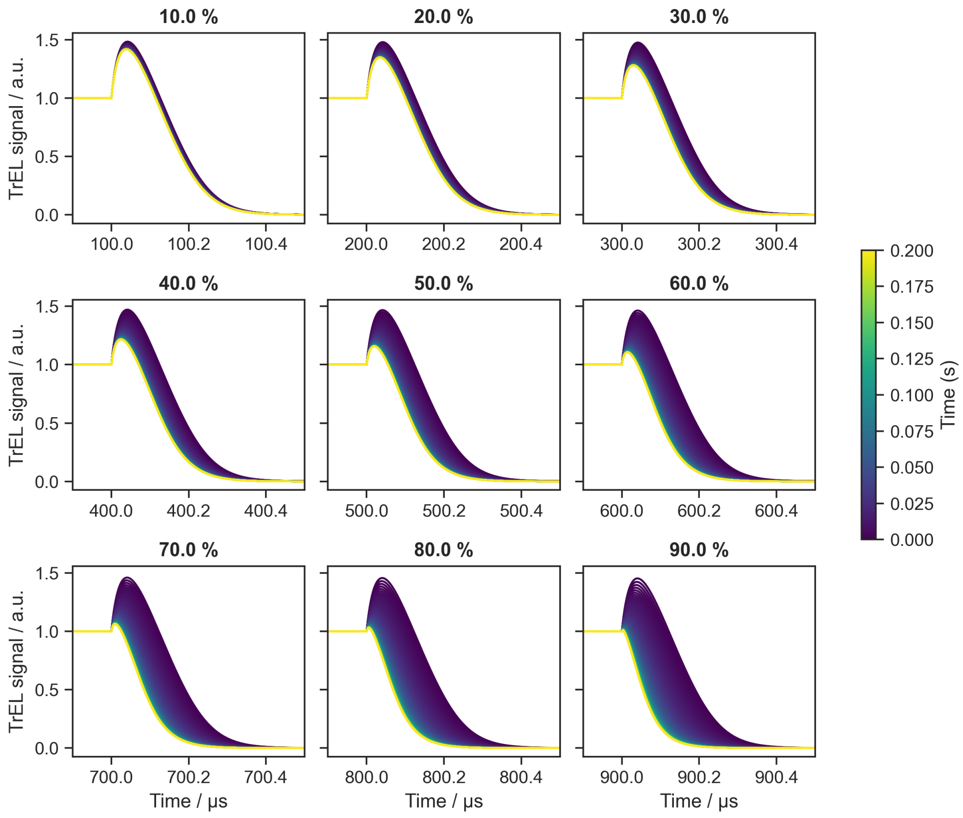

Figure SI 2: TrEL overshoot evolution during 200 1.7 V pulses at 1 kHz (0.2 s) for different duty cycles (%), normalised at TrEL plateau value.

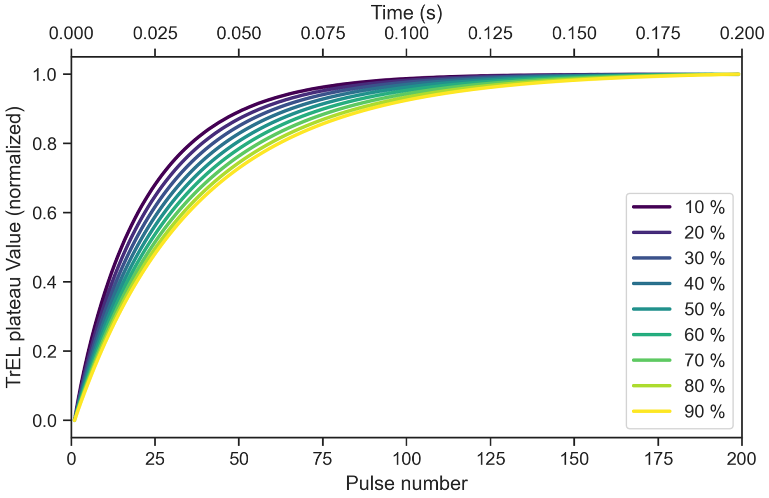

Figure SI 3: Normalized plateau value of TrEL signals for 200 pulses at 1 kHz, with varying duty cycles.

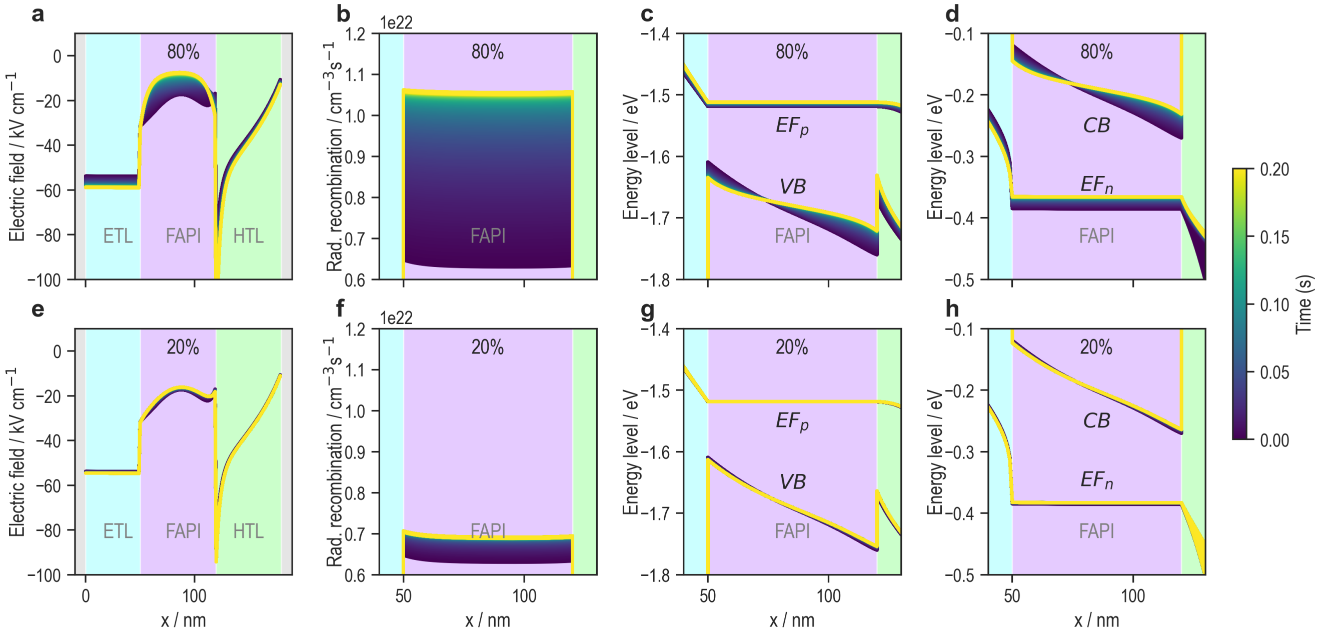

Figure SI 4: Temporal evolution of charge density profiles for simulated 1 kHz signal at 1.7 V, for 0.2 s (200 pulses). Dark blue color indicates the initial state, yellow the final state. Electric field (a,e) across the whole layer stack, band-to-band recombination (b,f), energy levels of VB and EF_p (c,g) and CB and EF_n (d,h) across the perovskite layer width (x). Top row (a)-(d) shows the temporal evolution for an 80% duty cycle, bottom row (e)-(h) for a 20% duty cycle.

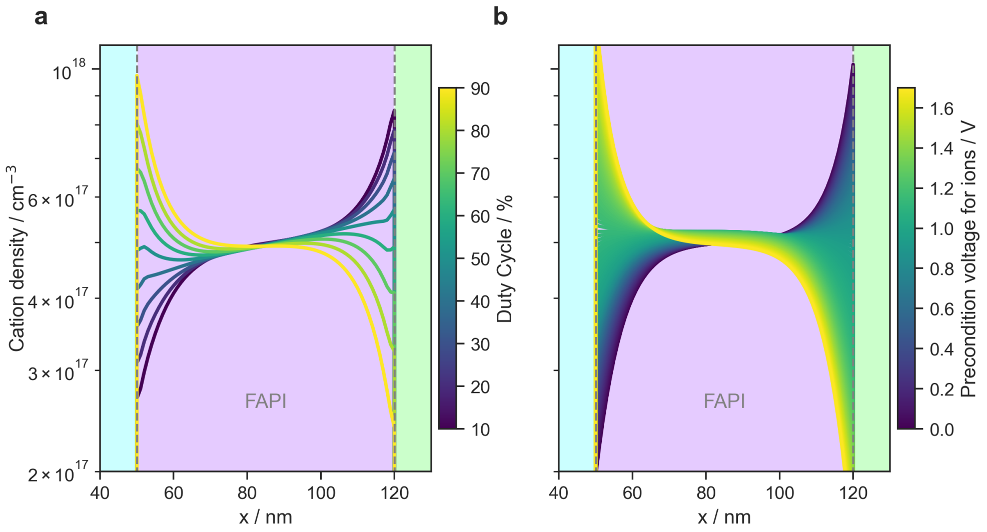

Figure SI 5: Mobile cation distribution profiles for (a) converged quasi-steady state from simulations under continuous voltage pulse train and (b) calculated for varying precondition voltages. Comparison allows to define which precondition voltages correspond to which duty cycles.

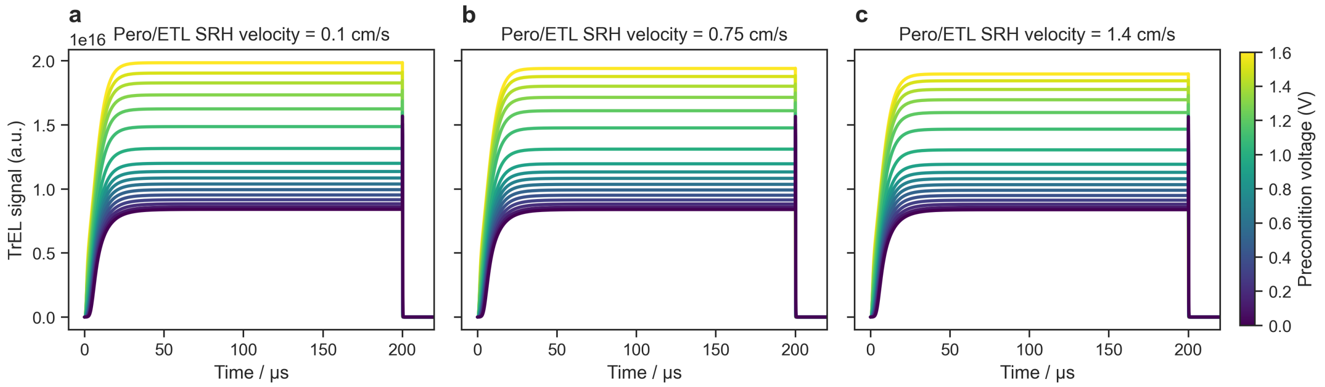

Figure SI 6: Simulated TrEL signals for a 200 µs pulse at 1.7 V for varying precondition voltages for the calculation of static ionic distributions. Interface SRH recombination velocity for electrons and holes at the perovskite/ETL interface is set to (a) 0.1 cm s^{-1}, (b) 0.75 cm s^{-1} and (c) 1.4 cm s^{-1}. Recombination velocity at the perovskite/HTL interface is fixed at 0.75 cm s^{-1}.

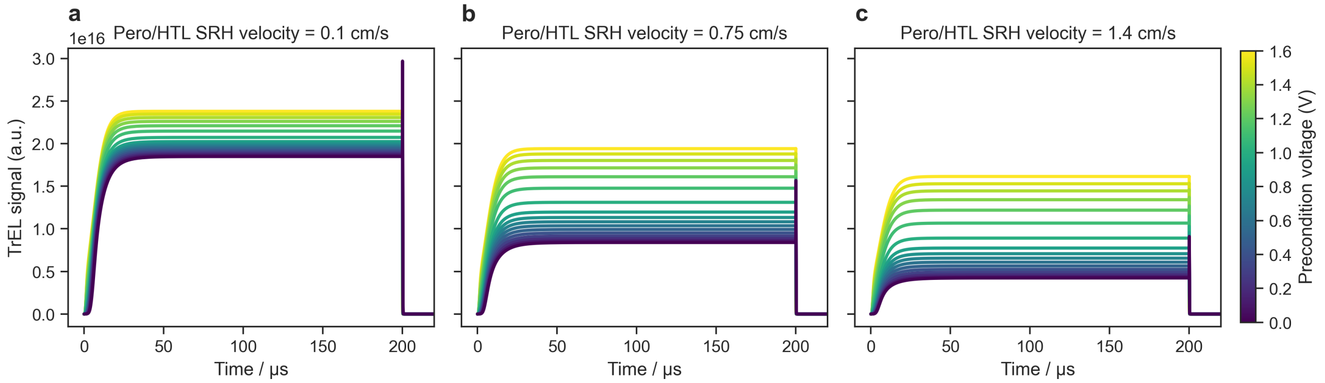

Figure SI 7: Simulated TrEL signals for a 200 µs pulse at 1.7 V for varying precondition voltages for the calculation of static ionic distributions. Interface SRH recombination velocity for electrons and holes at the perovskite/HTL interface is set to (a) 0.1 cm s^{-1}, (b) 0.75 cm s^{-1} and (c) 1.4 cm s^{-1}. Recombination velocity at the perovskite/ETL interface is fixed at 0.75 cm s^{-1}.

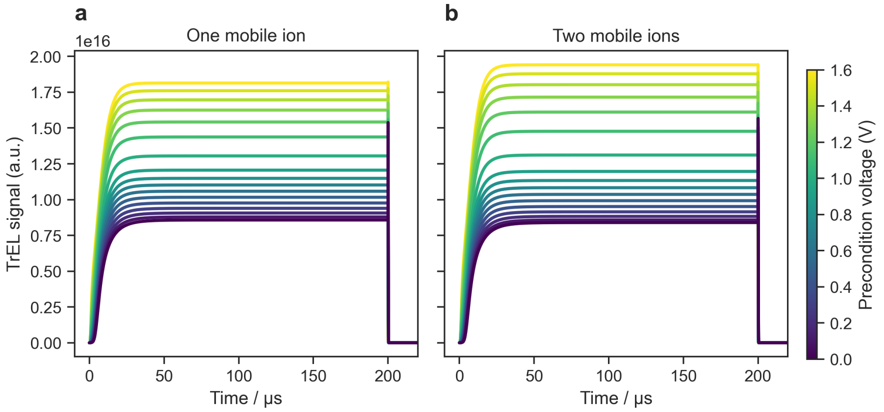

Figure SI 8: Simulated TrEL signals for a 200 µs pulse at 1.7 V for varying precondition voltages for the calculation of static ionic distributions. Anion and cation density are set to equal concentrations of $5 \cdot 10^{17}$ cm^{-3}. (a) Base-case where only cations are mobile and compensating anions are kept fixed in time and uniformly distributed throughout the perovskite layer. (b) Both cations and anions are allowed to redistribute according to the applied precondition voltage.

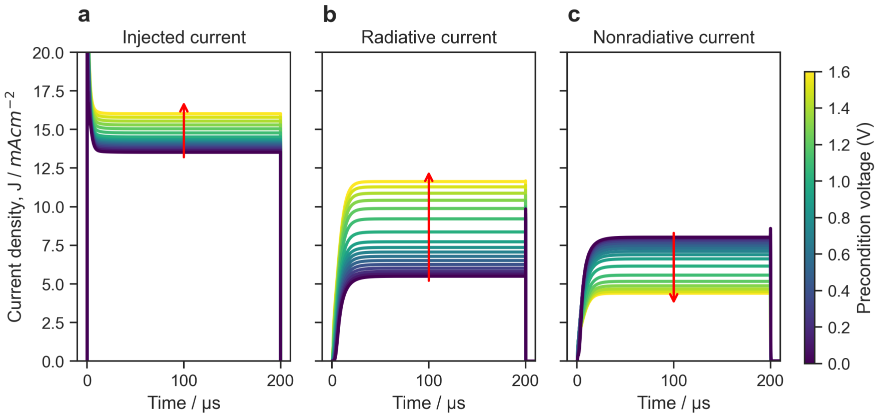

Figure SI 9: Current density transients (TrJ) for varying precondition voltages, where higher values represent the quasi-steady state at higher duty cycles. (a) Shows the total current in the device, (b) the current component from radiative band-to-band recombination and (c) the current component from non-radiative SRH recombination at the interfaces. The red arrows show the direction of the TrEL signal with increasing precondition voltage, representing different levels of ionic redistribution with increasing duty cycle.

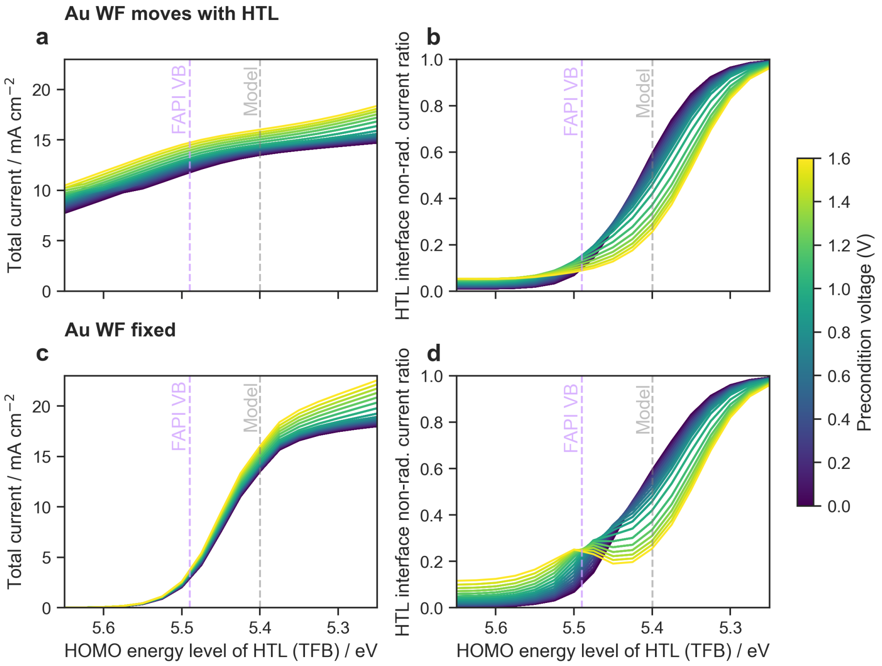

Figure SI 10: Current levels at the plateau regime, for a 1.7 V pulse, for varying injection barrier heights and precondition voltages, where higher precondition voltage values represent the quasi-steady state at higher duty cycles. The top row, (a) and (b), shows simulation results where the work function of the Au metal electrode moves as it is always kept 0.2 eV higher than the HOMO level of the HTL. The bottom row, (c) and (d), shows the case where the work function of Au is kept fixed at -5.2 eV. The total current density is shown in (a) and (c), the ratio of current loss at the perovskite/HTL interface to the total current is shown in (b) and (d), indicating an increase of interface recombination with increasing the injection barrier height. FAPI VB (purple dashed line) represents the valence band edge level used in simulations for the perovskite layer. The energy level used for the HTL is shown by the grey dashed line 'Model'.

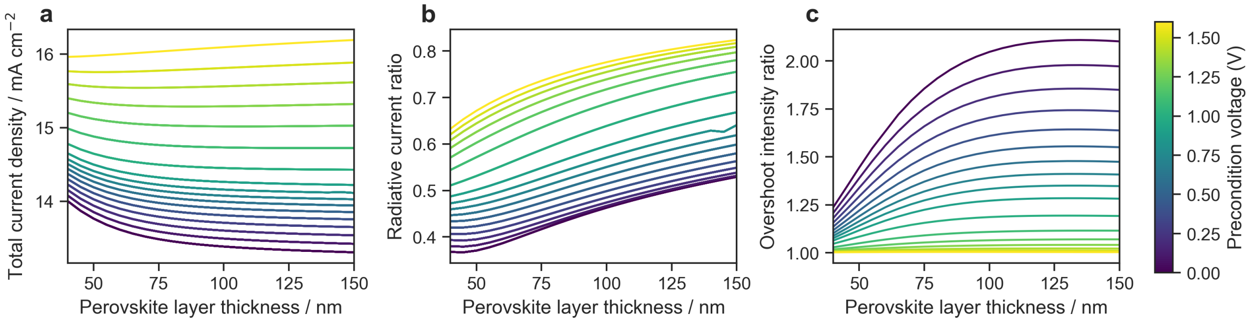

Figure SI 11: Simulation results for a 1.7 V pulse, for varying perovskite layer thickness and precondition voltages, where higher precondition voltage values represent the quasi-steady state at higher duty cycles. (a) Current density values at the plateau regime, (b) ratio of radiative recombination to total current density and (c) TrEL overshoot intensity relative to the plateau level.

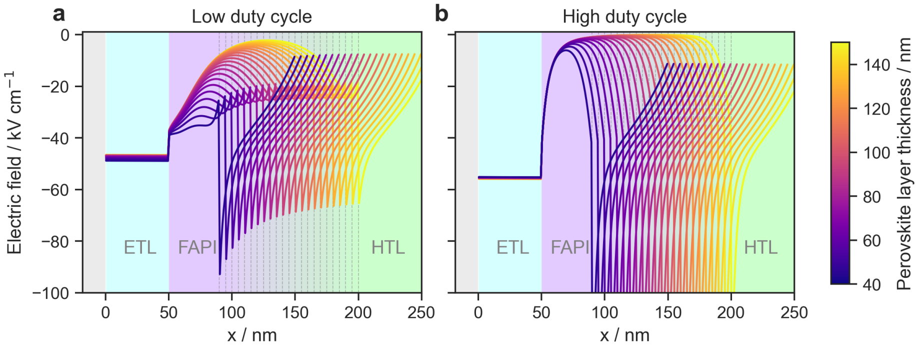

Figure SI 12: Electric field profiles across the device layer stack for a low duty cycle (0 V precondition voltage) (a) and for a high duty cycle (1.6 V precondition voltage) (b) for varying perovskite layer thickness.
